# Insights Into Virus‐Encoded RNA Silencing Suppressors Across Viral Families: A Focus on Viruses Infecting Solanaceae Crops

**DOI:** 10.1111/ppl.70840

**Published:** 2026-03-25

**Authors:** Saumik Basu, Sayanta Bera, Sourav Pal, Shirin Parizad, Pooja Malhotra, Trishita Ghosh, Clare L. Casteel, David W. Crowder

**Affiliations:** ^1^ Department of Entomology Washington State University Pullman Washington USA; ^2^ Department of Entomology University of Georgia Tifton Georgia USA; ^3^ Department of Microbiology, School of Life, Agricultural & Biotechnological Sciences Sister Nivedita University Kolkata West Bengal India; ^4^ Virginia Tech Southern Piedmont Agricultural Research and Extension Center Blackstone Virginia USA; ^5^ United States Department of Agriculture (USDA) Animal and Plant Health Inspection Service (APHIS), plant Protection and Quarantine (PPQ), plant Germplasm Quarantine Program (PGQP) Beltsville Maryland USA; ^6^ School of Integrative Plant Science, Plant Pathology and Plant‐Microbe Biology Section Cornell University Ithaca New York USA

**Keywords:** geminivirus, herbivores, pepper, phytohormones, potato, potyvirus, R‐gene, tomato, ubiquitin‐proteasome, viral suppressors

## Abstract

Viral suppressors of RNA silencing (VSRs) are proteins that interfere with antiviral defense mechanisms and enhance infection. For plant viruses, VSRs can be encoded in viral genomes and satellite molecules and play an important role in the virus's life cycle and in overcoming host defenses. However, a comprehensive review on the multifunctionality of VSRs and their role in the worldwide spread of plant viral diseases has not been performed. Here, we aim to synthesize the current understanding of the role of VSRs in the pathogenesis of Solanaceous plants, a family that includes many crops and medicinal plants. We focus on three key areas: (1) the diversity of VSRs and the mechanisms used to suppress antiviral defense, (2) the role of VSRs in viral pathogenesis beyond interfering with host RNA‐silencing, and (3) the coevolution between VSRs and plant host proteins. Additionally, we describe how VSRs promote the development of diseases by altering various steps in viral pathogenicity via induction of counter‐defense mechanisms. Specifically, a substantial body of evidence suggests that VSRs induce the suppression of antiviral silencing, abrogation of phytohormone signaling, and downregulation of R‐gene‐mediated host defense. Furthermore, we discuss how identifying and characterizing novel interactions between VSRs and Solanaceous host factors may be leveraged for developing sustainable pathogen and pest management strategies.

## 1 Introduction

Crop plants in the Solanaceae family are grown worldwide and include tomato ( *Solanum lycopersicum* ), potato ( *S. tuberosum* ), and pepper (*Capsicum annum*) (Olmstead et al. 2008; Gebhardt 2016). However, the productivity of crops in this family are threatened by over 40 viral genera (Haňcinský et al. 2020). Plant viruses infecting Solanaceous hosts include species of *Begomovirus* (e.g., tomato yellow leaf curl virus, TYLCV), *Potyvirus* (e.g., potato virus Y, PVY), *Tospovirus* (e.g., tomato spotted wilt virus, TSWV), *Nepovirus* (e.g., tomato ring spot virus, TRSV), *Tombusvirus* (e.g., tomato bushy stunt virus, TBSV), *Tobamovirus* (e.g., tomato mosaic virus, ToMV), and *Cucumoviruses* (e.g., tomato aspermy virus, TAV; cucumber mosaic virus, CMV). In response to these pathogens, Solanaceous plants have evolved multi‐layered defenses including RNA silencing, viral degradation, and phytohormone‐mediated defense (Teixeira et al. 2021). However, RNA silencing is the primary and critical antiviral strategy that has been shown to be conserved across nearly all crop species.

**FIGURE 1:**
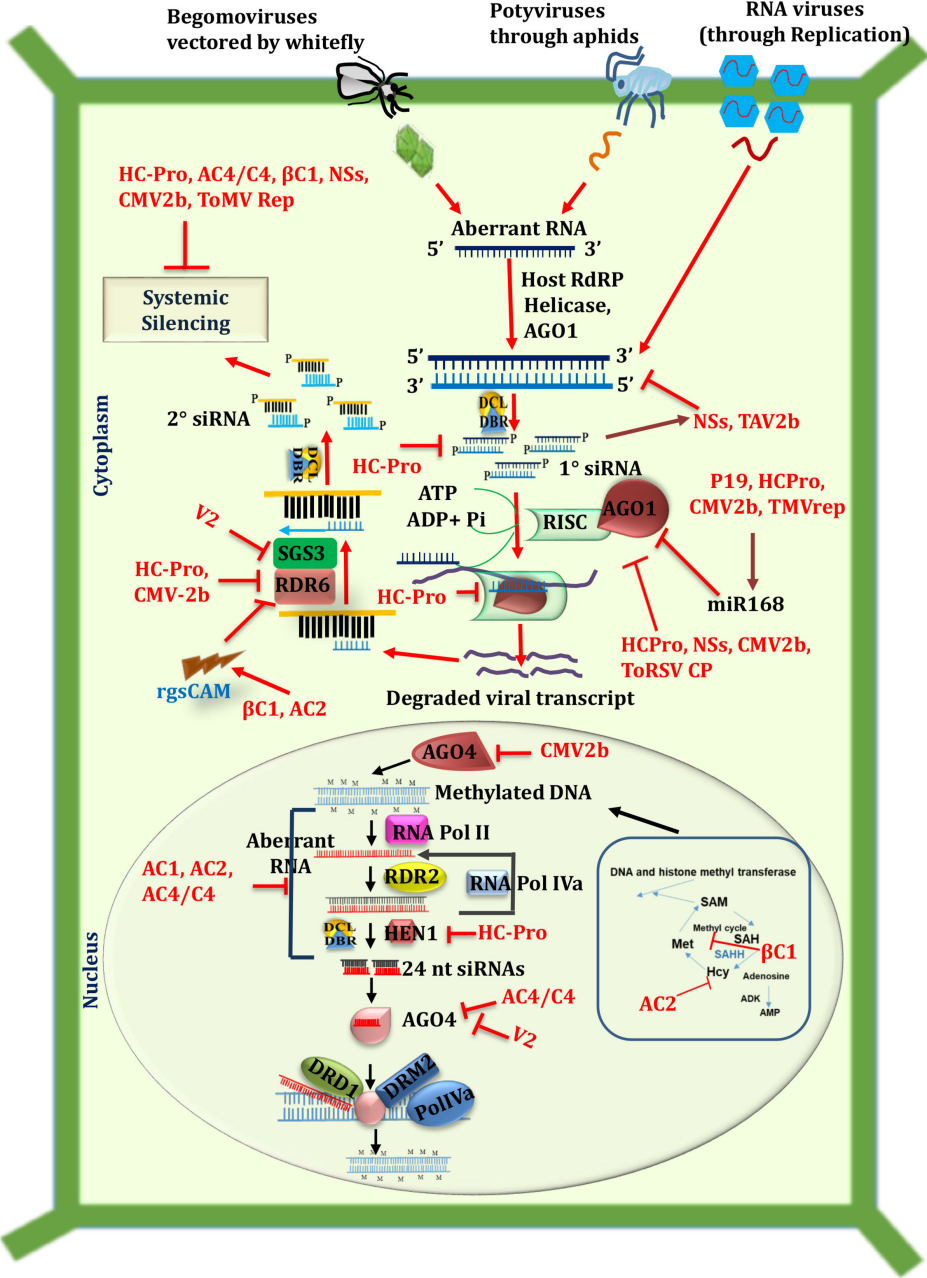
Antiviral RNA silencing pathways in tomato, depicting three unique silencing pathways: (1) Posttranscriptional gene silencing (PTGS) for degradation of viral mRNAs, (2) siRNA‐directed methylation leading to transcriptional gene silencing (TGS) of the methylated DNA, and (3) Endogenous mRNA silencing by miRNAs. The figure also depicts the multiple mechanisms by which VSRs have evolved to suppress host‐induced gene silencing.

The mechanism of antiviral RNA silencing begins with the detection and cleavage of viral double‐stranded RNA (dsRNAs) by dicer‐like proteins DCL 4 and DCL 2 into small interfering RNAs (siRNAs) (Baulcombe 2004). After this, the small interfering RNAs undergo methylation by the RNA methyltransferase Hua enhancer, with one strand integrated into the RNA‐induced silencing complex (RISC) with the assistance of heat shock protein 90 and other factors; the other strand is degraded. The viral small interfering RNA‐programmed RISC then binds to and cleaves viral transcripts based on sequence complementarity. Cleavage products are recognized by RNA‐dependent RNA polymerases, transcribed into new double‐stranded RNAs, and processed into secondary viral small interfering RNAs, further enhancing antiviral RNA silencing (Baulcombe 2004). While they act as a defense against viruses, RNA silencing also regulates gene expression related to plant growth and development, mediated by microRNA. MicroRNAs are generated by the same mechanism as viral small interfering RNA, but the process starts in the nucleus (Liu and Chen 2016; Liu et al. 2017). Consequently, host RNA silencing can be grouped into at least three partially overlapping pathways: (1) small interfering RNA‐mediated cytoplasmic gene silencing (known as posttranscriptional gene silencing, PTGS), (2) microRNA‐mediated silencing that regulates messenger RNA expression, and (3) DNA methylation‐dependent gene silencing (transcriptional gene silencing, TGS) (Baulcombe 2004) (Figure 1). These defenses have evolved throughout the plant kingdom to promote resistance to pathogens.

To cause successful infections, viruses must encode proteins that counteract defensive plant responses along with other proteins required for viral replication, translation, and movement (Basu et al. 2014; Csorba et al. 2015; Cheng and Wang 2017; Islam et al. 2019; Wu et al. 2019; Ziegler‐Graff 2020). Viral proteins that specifically interfere with RNA silencing‐based host defenses are called suppressors of RNA silencing (VSRs). A recent study demonstrated that VSR proteins may have radiated from 30 K viral movement protein, which evolved from the duplication of a capsid protein early in the evolution of plant viruses (Butkovic et al. 2023; Legarda et al. 2024; Ying et al. 2024). Thus, viruses use strategies for example, duplication and exaptation, to maximize the multi‐functionality of viral protein due to their limitation of small genome size.

The diversity and multi‐functionality of VSRs of viruses that infect Solanaceous plants have not been yet extensively reviewed. Here we address these knowledge gaps by reviewing: (1) VSR diversity and the mechanisms they use to suppress antiviral defense, (2) the role of VSRs in viral pathogenesis, and (3) coevolutionary dynamics mediated by VSRs. We then highlight research needs related to VSRs of viruses infecting Solanaceous crops, and research that could aid in improving the management of viral diseases by manipulating signaling pathways related to resistance. We focus our review first on VSRs encoded by four genera that include pathogens with a broad host range in Solanaceae (Table 1): (1) *Begomoviruses*, (2) *Potyviruses*, (3) *Cucumovirus*, and (4) *Orthotospovirus*. We then focus on VSRs encoded by three genera that have a narrow host range in Solanaceae and are often challenging to manage: (1) *Nepovirus*, (2) *Tobamovirus*, and (3) *Tombusvirus* (Kubota et al. 2003; Feng et al. 2011; Ghoshal and Sanfaçon 2014; Ocampo Ocampo et al. 2016; Bera et al. 2017; Basu et al. 2018; Bera et al. 2018; Gnanasekaran et al. 2019; Table 1). Overall, our review identifies key functions of VSRs and how these functions may aid in the future management of plant disease.

**TABLE 1:** List of different virus‐encoded silencing suppressors modulating various functions related to pathogenicity in Solanaceae crops.

Viral silencing suppressor	Virus genus	References
Counter defense (JA‐defense, SA‐defense, RNA decay etc.)		
V2/AV2	Begomovirus	Roshan et al. (2020)
AC2/AL2/C2	Begomovirus	Guerrero et al. (2020)
βC1	Begomovirus	Gnanasekaran et al. (2019)
NSs	Tospovirus	Wu and Ye (2020) and Du et al. (2020)
Coat protein	Nepovirus	Jovel et al. (2007)
HC‐Pro	Potyvirus	Endres et al. (2010) and Yang, Meng, et al. (2020)
2b	Cucumovirus	Jeon et al. (2017) and Ziegler‐Graff (2020)
P1	Poacevirus/Tiritmovirus	Tatineni et al. (2012)
Cell–cell and systemic movement		
V2/AV2	Begomovirus	Zhao et al. (2020)
βC1	Begomovirus	Gnanasekaran et al. (2019)
HC‐Pro	Potyvirus	Pollari et al. (2020)
VPg	Potyvirus	Eskelin et al. (2011) and Du et al. (2020)
2b	Cucomovirus	Nemes et al. (2014) and Shi et al. (2003)
Symptoms		
AC2/AL2/C2	Begomovirus	Matić et al. (2016) and Guerrero et al. (2020)
C4/AC4	Begomovirus	Fondong et al. (2007) and Rosas‐Diaz et al. (2018)
Rep	Tobamovirus	Ishibashi et al. (2010) and Sun et al. (2013)
HC‐Pro	Potyvirus	De et al. (2018) and Mäkinen and De (2019)
P19	Tombusvirus	Hsieh et al. (2009)
NSs	Tospovirus	Garcia‐Ruiz et al. (2018)
Translation and protein functions		
VPg	Potyvirus	Moury and Verdin (2012) and Charron et al. (2008)
Replication		
Rep	Tobamovirus	Ishibashi et al. (2010) and Sun et al. (2013)
Vector performance/transmission		
βC1	Begomovirus	Zhao et al. (2019)
NSs	Tospovirus	Wu et al. (2019)
HC‐Pro	Potyvirus	Dombrovsky et al. (2007)
2b	Cucumovirus	Mauck et al. (2010) and Ziebell et al. (2011)

## 2 Diversity and Mechanism of VSRs of Viruses Infecting Solanaceous Plants

### 2.1 VSRs Encoded by *Begomovirus*

*Begomovirus* species are whitefly‐transmitted ssDNA viruses in the family *Geminiviridae* that consist of circular single‐stranded DNA genomes (2.5–3 kb) with overlapping open reading frames. *Begomovirus* species encode multiple structurally and functionally different VSR proteins that suppress host gene silencing (Rojas et al. 2001; Zrachya et al. 2007; Shukla et al. 2013; Table S1). For example, TYLCV V2 inhibits SUPPRESSOR OF GENE SILENCING 3 protein (SGS3), a cofactor of RDR6 in PTGS and a key component of the host viral silencing machinery (Glick et al. 2008; Kumakura et al. 2009). In contrast, tomato yellow leaf curl China virus (TYLCCNV) V2 suppresses RNA silencing by sequestering siRNA molecules and inhibiting methylation‐mediated gene silencing, a part of transcriptional gene silencing pathways (Zhang et al. 2012; Wang et al. 2014, 2018, 2019). Similar to V2, VSRs such as AC4 and C4 also function as transcriptional and posttranscriptional gene silencing suppressors that interact with single‐stranded si‐/miRNAs (Chellappan, Vanitharani, and Fauquet 2005; Chellappan, Vanitharani, Ogbe, and Fauquet 2005) or AGO4 (Vinutha et al. 2018). The siRNAs are crucial components of the RNA silencing machinery. By binding to these siRNAs, AC4 prevents them from guiding the RISC to target viral RNA for degradation (Carluccio et al. 2018). VSRs also serve as transcriptional activators of viral and host genes to suppress transcriptional and posttranscriptional gene silencing (Dong et al. 2003; Wang et al. 2003, 2005; Luna et al. 2012; Jackel et al. 2015). For example, AC2 of tomato leaf curl virus (ToLCV) aids in silencing suppression by blocking histone methyltransferase and adenosine kinase, resulting in less methylation or by suppressing plant defense machinery (Castillo‐González et al. 2015; Ramesh et al. 2017; Basu et al. 2018). AL2, encoded by tomato golden mosaic virus (TGMV), induces calmodulin‐like protein (rgsCaM), resulting in autophagic degradation of SGS3 and suppressing the RNA silencing machinery (Yong Chung, Lacatus, and Sunter, 2014).

### 2.2 VSRs Encoded by *Potyvirus*

Potyviruses (family *Potyviridae*) are aphid‐transmitted positive sense RNA viruses (Revers and García 2015) that cause epidemic outbreaks in several crops (Parizad et al. 2017, 2018, 2019; Moratalla‐López et al. 2021; Movi et al. 2022). The viral genome length is approximately 10,000 bp, capable of coding a polyprotein that can cleave itself into 10 mature proteins; additionally, a small protein, P3N‐PIPO, is produced via a transcriptional frameshift. A nonstructural protein, HC‐Pro, was the first VSR identified encoded by a potyvirus (Table 1). HC‐Pro targets RNA silencing pathways by binding to virus‐derived siRNA (Kasschau and Carrington 1998; Del Toro et al. 2014; Del Toro et al. 2017). HC‐Pro also regulates AGO1 function by inducing miR168, a microRNA that targets AGO mRNA for degradation (Várallyay and Havelda 2013).

Aside from HC‐Pro, VPg acts as a VSR for potyviruses. VPg also interacts with SGS3, the cofactor of RDR6, to initiate its degradation by the proteasome and autophagy pathway (Cheng and Wang 2017); this interaction appears to be evolutionarily conserved across the *Potyviridae* (Rajamäki et al. 2014; Cheng and Wang 2017).

Some viruses, such as wheat streak mosaic virus (WSMV), triticum mosaic virus (TriMV), rice yellow mottle virus (RYMV), sugarcane streak mosaic virus (SCSMV), and wheat yellow mosaic virus (WYMV) from *Potyviridae* family, encode P1 protein that interferes with the plant's RNA silencing machinery, allowing the virus to evade the host's defense and replicate successfully (Tatineni et al. 2012). It is not well understood how P1 suppresses host defense, but research demonstrated that P1 destabilizes proteins involved in RNA silencing or interferes with the processing of small RNAs (Adrian Valli et al. 2006).

### 2.3 VSRs Encoded by *Cucumovirus*

Cucumoviruses from *Bromoviridae* like CMV have segmented, tripartite linear, positive sense ssRNA genomes comprised of RNA1 (3.4 kb), RNA2 (3.1 kb), and RNA3 (2.2 kb), each of which has a 32 tRNA‐like structure and a 52 cap and can be transmitted by over 80 species of aphids. The CMV 2b protein encoded by RNA2 binds strongly to host‐derived siRNA duplexes (e.g., miR171) and efficiently suppresses RDR6‐mediated posttranscriptional gene silencing (Diaz‐Pendon et al. 2007; Goto et al. 2007; Ye et al. 2009; Wang et al. 2011). CMV 2b also interacts with various protein components of RNA silencing machinery, such as AGO1 and AGO4 (Baumberger and Baulcombe 2005; González et al. 2010; Harvey et al. 2011; Duan et al. 2012; Hamera et al. 2012) (Figure 1). CMV 2b protein blocks AGO1 mediated cleavage associated with both miRNA and siRNA pathways (Zhang et al. 2006) and suppresses AGO4 mediated systemic silencing and DNA methylation (Ye et al. 2009). CMV 2b was further reported to decrease accumulation of 21–24 nt vsiRNAs generated by DCL4, DCL2, and DCL3 through RDR1‐dependent non‐cell‐autonomous antiviral silencing (Diaz‐Pendon et al. 2007). Besides CMV 2b, TAV encoded 2b protein suppresses posttranscriptional gene silencing by directly binding to siRNA duplexes (Chen et al. 2008). TAV 2b was also found to suppress the accumulation of both 52 secondary siRNAs and host RDR6‐specific mRNAs but has no control over the regulation of 32 secondary siRNAs (Zhang et al. 2008).

### 2.4 VSRs Encoded by *Tospovirus*

TSWV from *Tospoviridae* is a devastating tospovirus with a genome containing three negative‐sense ssRNA (Margaria and Rosa 2015), which is transmitted through several species of thrips insects. Nonstructural proteins (NSs) encoded by viral RNA (Parrella et al. 2003) block the antiviral silencing by binding with dsRNA in a size‐independent manner or by interacting with SGS3 (Chen et al. 2022). Unlike other VSRs, NSs exhibit antiviral silencing in a dose‐dependent manner; an increase of NSs directly correlates with higher inhibition of host silencing machinery (Takeda et al. 2002; Bucher et al. 2003; Hedil et al. 2015; Ocampo Ocampo et al. 2016). TSWV NSs can inactivate RNA silencing by interacting with small and long dsRNAs (ds‐miRNA and ‐siRNA precursors) through the dsRNA‐binding motif and interfering with their cleavage by Dicer‐like nucleases and uploading into RISC (Schnettler et al. 2010). TSWV NSs also bind AGO1 to prevent its sequestering of siRNAs (Giner et al. 2010; Hedil et al. 2015). Besides TSWV, NSs of another tospovirus, tomato yellow ring virus (TYRV), also block local and systemic silencing and sequester both long and short double‐stranded RNAs (Hedil et al. 2015). Unlike TSWV, TYRV NSs exhibit a distinct mechanism of silencing suppression; it possesses NTPase/phosphatase activity that may inhibit PTGS by removing the 5′ phosphate from dsRNA (the substrate for Dicer), thereby preventing siRNA biogenesis. Notably, despite being expressed at significantly lower levels than other tospoviral VSRs, TYRV NSs remain a potent suppressor of systemic silencing (Hedil et al. 2015). Recent insights suggest that this high efficiency may also involve the ability of NSs to exert VSR activity by interacting with and destabilizing AGO1 and reducing its steady‐state levels, thus neutralizing the core machinery of the host's antiviral response (Hedil et al. 2015).

### 2.5 VSRs Encoded by Other Viral Genera

TRSV is transmitted by a nematode, *Xiphinema americanum*, and belongs to the family *Secoviridae* (Genus: *Nepovirus*) (Brown et al. 1993). The genome consists of bipartite ssRNAs that encode two polypeptides and are cleaved by proteases. TRSV coat protein exhibits VSR activity by interacting with AGO1 and destabilizing it by reducing its steady state level (Karran and Sanfaçon 2014). TRSV X4 protein (encoded by RNA2) has a diverse sequence across nepovirus species and has been reported to have silencing suppressor activity in some TRSV species (Jafarpour and Sanfaçon 2009).

TBSV from the tombusvirus genus (family: *Tombusviridae*) has a (+) ssRNA genome (4.8 kb) with five open reading frames and is passively transmitted by wind and by mites, aphids, and the fungus *Olpidium brassicae. TBSV* encodes the P19 protein, which sequesters siRNA duplexes of specific size with high affinity, particularly 21 nt ds siRNA with 2 nt, 3′ overhangs (Hsieh et al. 2009; Danielson and Pezacki 2013). Because of its unique small RNA‐ligand binding property, this protein prevents entry of the specific siRNA into the RISC by competing with AGO1, but fails to destabilize programmed RISC (Silhavy et al. 2002; Lakatos et al. 2006; Figure 1).

P19 has a strong affinity for DCL4 (Dunoyer et al. 2005; Deleris et al. 2006) and in addition to siRNA duplexes, P19 also has high affinity for miRNA duplexes of 23 nt (Chapman et al. 2004; Chen et al. 2008; Nasheri et al. 2011). P19 can adopt alternative strategies to suppress RNA silencing in hosts. For example, expression of P19 during infection induces the host miRNA, miR168, which downregulates AGO1 (Várallyay et al. 2010; Figure 1A,B). Because of the ability of P19 to sequester small RNA duplexes, it is a tool for capturing small RNAs in various heterologous systems with more complex RNA silencing pathways (Danielson and Pezacki 2013).

ToMV from *Virgaviridae* encodes a replication‐associated protein (Rep) that can suppress posttranscriptional gene silencing (Kubota et al. 2003). However, while ToMV Rep inhibited posttranscriptional gene silencing in inoculated leaves, Rep failed to suppress ToMV‐specific posttranscriptional gene silencing in hosts that already had established infections (Kubota et al. 2003). Thus, ToMV Rep suppresses the use of viral‐specific small RNAs and makes them unavailable for being used for the homology‐dependent cleavage of ToMV RNA (Tamai et al. 2010; Figure 1).

In summary, the common feature of all VSRs is their ability to interfere with the host's RNA silencing machinery. Despite originating from different virus families, VSRs share the common goal of suppressing the host's RNA silencing‐mediated antiviral defense mechanisms. They achieve this by targeting various components of the host's small RNA pathways, such as miRNAs and siRNAs, and disrupting the function of RISCs. Additionally, VSRs often interact with key proteins involved in RNA silencing, such as DCL, AGO1, AGO4, and RDR6, to inhibit their activity and prevent the degradation of viral RNA. Thus, the commonality lies in their role as molecular tools that viruses use to counteract the host's RNA silencing‐based immune responses.

## 3 Role of VSRs in Pathogenesis

Successful pathogenesis occurs when a virus overcomes host defenses, replicates, and spreads through the plant and to the next host (Mandadi and Scholthof 2013; García and Pallás 2015). VSRs can also play an important role in multiple aspects of pathogenesis due to multi‐functionality (García and Pallás 2015). In this section (Table 1), we highlight various ways VSRs can promote pathogenesis in addition to suppressing RNA silencing and identify critical research gaps on the multifunctionality of VSRs.

### 3.1 VSR Interacts With Host RNA, Proteins and Regulates Phytohormones to Alter Plant Development and Immunity

#### 3.1.1 Begomovirus

The N‐terminal of tomato leaf curl Java virus (ToLCJV) V2 protein contains nuclear export signals that facilitate viral movement from the nucleus to the plasmodesmata (Sharma et al. 2011), possibly via interacting with EXPORTIN‐α and V1 (Zhao et al. 2020), while the C‐terminal affects viral pathogenicity and hypersensitive response (Sharma and Ikegami 2010). TYLCV V2 aggregates to bind viral DNA molecules for nucleo‐cytoplasmic shuttling, which drives viral infection (Moshe et al. 2015). TYLCV V2 protein interacts with papain‐like cysteine proteases, disrupting their ability to trigger host defenses (Bar‐Ziv et al. 2012; Roshan et al. 2018).

Tomato leaf curl New Delhi virus (ToLCNDV) AC2 protein alters the regulation of host miRNAs that control transcription factors involved in tomato development processes (Kumar and Naqvi 2016). ToLCNDV AC2 also suppresses hypersensitive response in both tomato and *N. benthamiana* (Hussain et al. 2007). AL2 protein encoded by TGMV interacts and inactivates host SUCROSE NON‐FERMENTING1 (SNF1)‐RELATED KINASE 1 (SnRK1) and ADENOSINE KINASE (ADK), responsible for viral genome methylation, an epigenetic defense against TGMV (Wang et al. 2003, 2005; Raja et al. 2008). Both SnRK1 and ADK are important host factors that maintain host methylation cycles through regulation of host metabolism and S‐adenosyl methionine (SAM)‐dependent methylation, respectively.

The sequence of C4 gene exhibits variability across the geminivirus family. C4 of ToLCV, when expressed constitutively, can induce symptom expression (Rigden et al. 1994; Krake et al. 1998). Beet curly top virus (BCTV) C4, which shares no sequence homology with ToLCV C4, can function as a pathogenicity determinant and contribute to enhanced phloem cell division and elongation (Pooma and Petty 1996; Latham et al. 1997). The presence of conserved N‐myristoylation domains in AC4 proteins determines their membrane binding, pathogenicity and disease symptom expression (Fondong et al. 2007; Rosas‐Diaz et al. 2018). AC4 also interacts with the receptor‐like kinase BARELY ANY MERISTEM 1 (BAM1), which is involved in the cell‐to‐cell movement of RNA silencing signals. This interaction helps AC4 to hinder the spread of the silencing signal, thereby suppressing the plant's defense response (Carluccio et al. 2018).

Transgenic overexpression of TYLCCNV βC1 also induces developmental abnormalities in leaves by decreasing miR165/166 levels and by enhancing the transcription factors that are responsible for maintaining abaxial and adaxial leaf polarity (Yang et al. 2008). βC1 also suppresses methylation through interaction and inactivation of S‐adenosyl homocysteine hydrolase, an essential enzyme involved in the methyl cycle (Yang et al. 2011).

Geminiviral suppressors obstruct phytohormone biosynthesis or signaling pathways that are necessary to regulate homeostatic balance between growth and virus‐induced stress in plants. For example, the C2 protein of TYLCV interacts through an evolutionarily conserved mechanism with the ubiquitination domain of RPS27A (a ribosomal protein) and inhibits the degradation of JAZ1 protein, which represses jasmonic acid signaling and terpene biosynthesis (Luan et al. 2013). Similarly, TYLCV C2 protein interacts with the catalytic subunit of constitutive photomorphogenesis 9 signalosome multi‐subunit protein complex, affecting its ability to regulate E3 ubiquitin ligase and impair jasmonic acid signaling (Lozano‐Durán et al. 2011; Rosas‐Díaz et al. 2016). Thus, C2 of begomoviruses attenuates the jasmonic acid pathway through transcriptional repression of jasmonic acid‐responsive genes or interacting with ubiquitin and subverting JAZ1‐MYC‐mediated jasmonic acid response (Lozano‐Durán et al. 2011; Rosas‐Díaz et al. 2016; Li et al. 2019; Ziegler‐Graff 2020).

**FIGURE 2:**
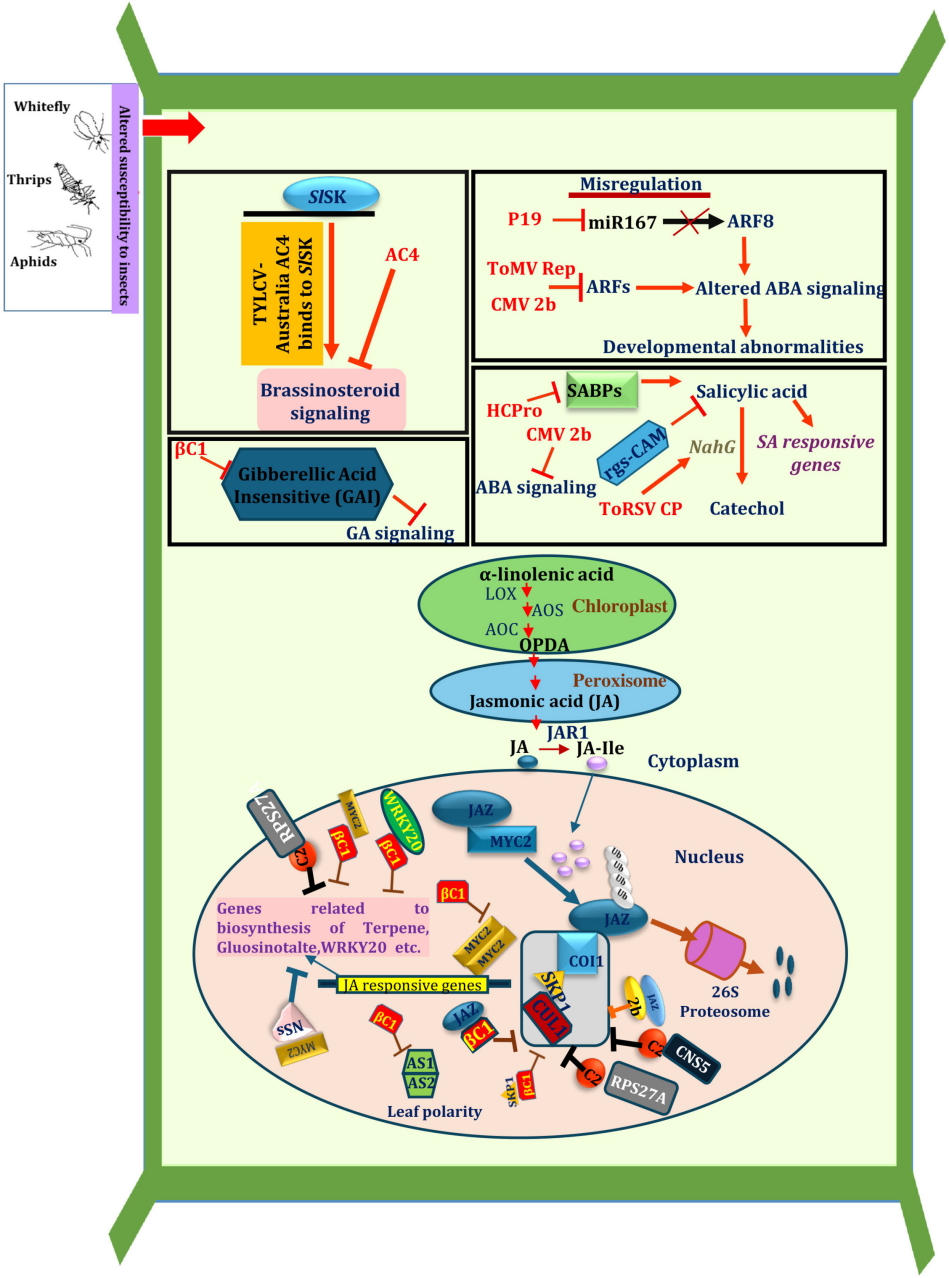
Schematic diagram showing roles of different VSRs in interfering with phytohormone signaling pathways.

Another plant hormone, brassinosteroid, is associated with other phytohormones to promote plant growth and defense (Belkhadir and Jaillais 2015). However, AC4 from ToLCV‐Australia interacts with a novel shaggy‐like kinase in tomato (SlSK) through 12 amino acids present in the C‐terminal to interfere with brassinosteroid signaling (Piroux et al. 2007; Dogra et al. 2009; Figure 2B). TYLCCNV βC1 also interacts with asymmetric leaves (AS1) to attenuate jasmonic acid defense (Fu et al. 2007; Yang et al. 2008; Figure 2A). TYLCCNV βC1 can also interact with helix–loop–helix transcription factors to reduce terpene and glucosinolate biosynthesis and impact phytohormones levels (Li et al. 2014; Figure 2A).

#### 3.1.2 Potyvirus

HC‐Pro uses autoproteolytic activity to cleave the viral polyprotein through its C‐terminus and initiate viral infection (Carrington et al. 1989). Recently, AGO1 was shown to be recruited by HC‐Pro, which causes the production of stable virus particles and results in systemic infection (Pollari et al. 2020). HC‐Pro is also active in specific protein–protein and protein–RNA interactions that affect plant metabolism and virus multiplication (Whitham and Wang 2004; Du et al. 2011). Evidence reveals HC‐Pro interacts with the plant's cytoplasmic EXORIBONUCLEASE 4 (Xrn4), another major cellular antiviral mechanism involved in RNA decay and VSR activity (Li and Wang 2018). HC‐Pro also causes viral symptoms by inducing the production of reactive oxygen species and by reducing antioxidant accumulation (Ivanov et al. 2016; De et al. 2018; Mäkinen and De 2019). Recently, Yang, Qiu, et al. (2020) showed HC‐Pro directly interacts with CATALASE 1 (CAT1) and CATALASE 3 (CAT3) in the cytoplasm of tobacco plants (Table S1). As a result, H_2_O_2_ was produced to help with viral infection, and a reactive oxygen burst induced systemic cell death in infected plants. Similarly, VPg, apart from acting as a VSR, plays a role in viral translation and systemic movement (Eskelin et al. 2011). A recent article demonstrated that a multiprotein complex consisting of HC‐Pro and VPg recruits a host protein, varicose, which assists in initiating systemic infection in the plant (De et al. 2020).

HC‐Pro suppresses salicylic acid‐mediated defense signaling by interacting with an *Arabidopsis thaliana* homologue of SALICYLIC ACID‐BINDING PROTEIN 3 (SABP3). By limiting the production of salicylic acid, HC‐Pro weakens host defenses to facilitate viral infection (Poque et al. 2018). However, another study with the HC‐Pro of tobacco vein banding mosaic virus showed the induction of salicylic acid and associated host defense response (Yang et al. 2016). From these studies, it can be concluded that the function of salicylic acid in potyvirus infection is dependent on specific plant‐potyvirus interactions. A host‐encoded silencing suppressor found in *Chenopodium quinoa* (CqCA1) also interacts with HC‐Pro (Poque et al. 2018), suggesting a conserved interaction. Further, HC‐Pro affects jasmonic acid‐regulated gene expression in plants (Endres et al. 2010). Apart from salicylic acid and jasmonic acid, HC‐Pro was also found to induce auxin accumulation in plants, leading to abnormal growth (Yang, Meng, et al. 2020).

#### 3.1.3 Cucomovirus

CMV 2b protein plays a role in systemic and small viral movements (Ziebell et al. 2011; Zhou et al. 2014). Stabilization of the C‐terminal of CMV 2b protein maintains the CMV2b‐siRNA‐ribonucleoprotein complex structure that is necessary for infectivity and viral spread (Gellért et al. 2012). Alanine scanning mutagenesis has identified conserved amino acid residues in CMV 2b protein that were responsible for cell‐to‐cell and long‐distance movement and silencing suppressor activity (Nemes et al. 2014). There are also reports of interaction between CMV 2b and *Arabidopsis* CAT3 that causes necrotic spots in systemic leaves (Nakahara and Masuta 2014).

TAV 2b binds primarily to duplex siRNAs in a length‐specific manner and can also bind to miRNA duplexes and single‐stranded RNAs of various lengths (Rashid et al. 2008). The N‐terminal (12 amino acids) of the TAV 2b protein plays an essential role in recombination with 2b proteins of CMV 2b and is key for the systemic infection of host plants (Shi and Palukaitis 2011). The ability of the 2b protein of CMV and TAV to interact with miRNAs was also revealed by spatial and temporal changes in various miRNAs and their target mRNA expressions in response to viral infection in tomato (Feng et al. 2011). The CMV 2b protein promotes cell‐to‐cell movement of pseudorecombinant viruses and plays a vital role in hypersensitive cell death and virus resistance (Shi et al. 2003); these mechanisms were further demonstrated as both activities were abolished through mutation in the functional domain (Li et al. 1999).

During CMV infection, 2b protein binds to JAZ1 to inhibit degradation and induction of jasmonic acid (Ziegler‐Graff 2020; Figure 2A). Constitutive expression of Fny‐CMV 2b protein downregulates 90% of jasmonic acid‐regulated genes without affecting jasmonic acid biosynthesis (Lewsey et al. 2010). CMV 2b protein also interferes with salicylic acid signaling by interacting with rgs‐CaM (Ji and Ding 2001; Lewsey et al. 2010; Jeon et al. 2017; Figure 2B; Table S1). Wu et al. (2017) investigated how CMV 2b protein can repress host JAZ1 protein, a repressor for MYC transcription factors. In unstressed hosts, low levels of jasmonic acid favor JAZ1 accumulation and suppress jasmonic acid signals. However, under the influence of biotic stressors (herbivores), increased jasmonic acid levels facilitate the degradation of JAZ1 through the 26S proteasome machinery (Figure 2A).

#### 3.1.4 Tospovirus

VSR NSs play an important role in viral infection and movement within the Tospoviridae family (Takeda et al. 2002). This VSR also maintains pathogenicity in other heterologous viruses that are deficient in functional suppressors (Ocampo Ocampo et al. 2016). NSs also induce the development of systemic infection and TSWV induced symptoms through inhibition of host plant antiviral silencing (Garcia‐Ruiz et al. 2018). Similarly, TSWV NSs promote persistent infection and vector‐borne transmission by western flower thrips ( *Frankliniella occidentalis* ) (Margaria et al. 2014). Moreover, NSs interact with transcription factors to subvert jasmonic acid‐mediated defense against western flower thrip vectors.

#### 3.1.5 Tombusvirus

Various mutations in the siRNA binding site of P19 generate a multitude of symptoms in host plants that compromise systemic silencing, but mutations in other sites cause developmental defects (Hsieh et al. 2009). For example, the efficiency of RNA silencing is highly sensitive to how P19 interacts with siRNA duplexes. Mutations in the central region of the P19 binding surface typically result in more severe symptoms (stronger suppression) compared to mutations at the periphery, as the center is critical for sequestering the 21‐nucleotide siRNA duplex. The generation of P19 mutants, and symptoms expressed by mutants, are also dependent on host physiology due to compromised host‐dependent siRNA sequestration (Hsieh et al. 2009). P19 interferes with HEN‐1‐mediated methylation of miRNAs and decreases endogenous miRNA stability (Lózsa et al. 2008). P19 also interacts with uncharacterized plant RNA‐binding ALY proteins (involved in nucleo‐cytosolic mRNA transport and influencing growth and development of plant) through its RNA‐binding domain and alters the localization of ALY from nucleus to cytoplasm (Uhrig et al. 2004; Canto et al. 2006). Moreover, ectopic expression of the VSR TBSV P19 in hosts results in misregulation of miR167, which targets AUXIN RESPONSE FACTOR 8 (ARF 8) (Jay et al. 2011; Figure 2B; Table S1) and causes abnormalities.

#### 3.1.6 Tobamovirus

ToMV Rep plays an important role in the movement and encapsidation of the viral genome. In addition, Rep also interacts with host plant factors that drive symptom development (e.g., chloroplast ferredoxin I in tobacco, NAC domain transcription factors in *Arabidopsis*, and various other cellular proteins from tomato) (Ishibashi et al. 2010; Sun et al. 2013). Membrane‐bound Rep also plays a critical role in guanylation of nascent RNAs to form 5′ cap, which is required for the stability of nascent RNA undergoing elongation and protein synthesis.

ToMV infection in tomato induced levels of trans‐acting (ta)‐siRNAs that regulate the AUXIN RESPONSE FACTORS (ARF) (Yifhar et al. 2012; Figure 2B). ToRSV CP in tobacco induces NahG expression and breaks down salicylic acid into catechol (Figure 2B), which can result in increased lesion size, facilitating the spread of this virus efficiently and systemically (Jovel et al. 2011). Furthermore, salicylic acid has been reported to work upstream of siRNA pathway and amplify siRNA signaling in plants (Alazem and Lin 2015). VSRs play a vital role in tuning these interactions to facilitate viral infection. However, more studies need to be done to identify the protein/s that connects siRNA pathway to salicylic acid.

VSRs use conserved strategies to hijack plant pathways for infection, often inhibiting RNA silencing by binding to small RNAs and disrupting defense mechanisms. They manipulate phytohormone pathways such as JA and SA signaling to weaken immune responses, often through interactions with key regulators, including JAZ1 or rgs‐CaM. VSRs such as HC‐Pro and P19 also interact with host proteins to stabilize viral RNA or alter ROS levels, facilitating infection and systemic spread. In addition, VSRs can repress transcription factors or affect miRNA stability, thereby disrupting host growth and development. These conserved interactions underscore the sophisticated tactics VSRs use to subvert host immunity while aiding the virus in infecting plants.

### 3.2 Role of VSRs in Vector Transmission

VSRs play a role in altering the susceptibility of Solanaceous hosts to both vectors and nonvector herbivores by interfering with phytohormone signaling and volatiles emitted (Tungadi et al. 2017; Ziegler‐Graff 2020). In the following sections, we briefly discuss the role of VSRs in affecting vector fitness and behavior, and effects on pathogen transmission, building on a previous review (Ray and Casteel 2022).

#### 3.2.1 Begomovirus

βC1 interferes with the feeding behavior of the vector whitefly by interfering with three different host factors: AS1, MYC2, and SKP1 (Yang et al. 2008; Li et al. 2014; Jia et al. 2016; Figure 2A). Conversely, accumulation of βC1 in the phloem of infected hosts and binding with transcription factor WRKY20 deters nonvector herbivores, but favors whitefly vectors (Zhao et al. 2019).

βC1 favors herbivore insects by inhibiting glucosinolate‐mediated anti‐herbivore defense (Hopkins et al. 2009). Another monopartite VSR, C2, was reported to improve the performance of whiteflies by inhibiting jasmonic acid‐signaling and terpene biosynthesis by subverting ubiquitination (Luan et al. 2014; Rosas‐Díaz et al. 2016; Li et al. 2019; Figure 2A).

#### 3.2.2 Potyvirus

HC‐Pro acts as a bridge between virions and receptor proteins (stylins) in aphid stylets (Blanc et al. 1997; Dombrovsky et al. 2007; Webster et al. 2018). From the insect vectors' aspect, there is little information on the receptors that bind HC‐Pro (Dombrovsky et al. 2007). However, HC‐Pro manipulates aphid biology; PVY HC‐Pro in transgenic *N. benthamiana* was reported to enhance the growth of vector *Myzus persicae* (Westwood et al. 2014). In contrast, it has been revealed that transiently expressed HC‐Pro decreased aphid fecundity on *N. benthamiana* leaves (Casteel et al. 2014). Moreover, Casteel et al. (2014) also showed a decrease in aphid fecundity in the presence of ectopically expressed VPg protein.

#### 3.2.3 Cucomovirus

The 2b protein encoded by CMV induces the host plant to release a specific blend of volatiles that provides a favorable environment for both aphid vectors and the horizontal transmission of the virus (Lewsey et al. 2010; Wu et al. 2017). CMV 2b protein also facilitates aphid invasion on plants by inhibiting the AGO1‐mediated biosynthesis of an aphid‐repelling glucosinolate, 4‐methoxy‐indole‐3‐yl‐methylglucosinolate, and by interfering with AGO1 (Westwood et al. 2013). CMV 2b protein affects the attractiveness and fecundity of the vector green pea aphid ( *M. persicae* ) and indirectly affects virus transmission by suppressing jasmonic acid‐mediated defense signaling (Mauck et al. 2010; Ziebell et al. 2011).

#### 3.2.4 Other Virus Genera

Nonstructural proteins of TSWV alter the preference of the vector, western flower thrips, while increasing their reproductive fitness and developmental rate (Wu et al. 2019). TSWV NSs enhance plant attractiveness to thrips by interacting with various MYC transcription factors and inhibiting jasmonic acid‐signaling (Wu et al. 2019; Figure 2A). Besides thrips, TSWV‐infected plants have enhanced performance and fecundity to two‐spotted spider mite, *Tetranychus urticae* (Nachappa et al. 2013). Currently, it is not known if the coat protein (VSR) of *Nepovirus* affects the behavior of its vector.

## 4 Coevolutionary Dynamics Between VSRs and Host Proteins

VSRs are critical pathogenesis factors that coevolve with the plant and its defenses. To understand the drivers of VSR evolution, the gene‐for‐gene model can be implemented, as first described in the flax‐rust system (Flor 1955). In this model, the interaction between specific plant and viral factors can trigger an incompatible interaction, resulting in a hypersensitive response (HR) that limits infection (Fraile and García‐Arenal 2010). While this provides a fundamental framework for understanding direct recognition, it is increasingly clear that the Guard Hypothesis (van der Hoorn and Kamoun 2008) also explains many solanaceous defense responses. In the latter, *R* proteins monitor modifications to host targets rather than the VSRs themselves. However, to maintain focus on the direct molecular interactions between VSRs and the RNAi machinery, this review primarily utilizes the gene‐for‐gene framework. Regardless of the recognition model, the HR is often associated with an increase in salicylic acid, leading to localized cell death (Radojičić et al. 2018). Here, we highlight studies where plant factors directly or indirectly detect VSRs to trigger these defenses, as well as VSR‐independent counter‐defense strategies that allow viruses to function without inducing a host response.

### 4.1 Begomovirus

Begomoviral VSRs function as elicitors and pathogenicity determinants (Voorburg et al. 2020). For example, the V2 protein of ToLCJV, *Cotton leaf curl Kokhran virus*, and *Papaya leaf curl virus* elicit hypersensitive responses in *N. benthamiana* and tomato (Mubin et al. 2010; Sharma and Ikegami 2010). To counter the plant defense response, the C2 protein neutralizes the effect of the V2 protein and ensures efficient viral infection (Trinks et al. 2005; Mubin et al. 2010).

Another VSR protein, AC4, from different geminiviruses that infect a range of hosts, possesses a N‐myristoylation motif (conserved and consensus) responsible for membrane binding, elicitor of disease symptoms and pathogenicity determinant (Fondong et al. 2007, Van Wezel et al. 2002). AV2 protein of tomato leaf curl Palampur virus induces genes associated with salicylic acid‐signaling in tomato (Roshan et al. 2020). Moreover, some recent findings suggest a role of C2 from tomato yellow leaf curl‐Sardinia virus (TYLCSV) as a virulent factor that triggers a hypersensitive response in plants. The C2 protein of TYLCSV acts as a pathogenicity determinant and a 16‐amino acid domain is responsible for inducing a hypersensitive response in plants (Matić et al. 2016; Guerrero et al. 2020). Interestingly, the same study showed a lack of hypersensitive response during TYLCSV infection, suggesting the presence of some other viral protein that counterattacks the host defensive response (Matić et al. 2016).

### 4.2 Potyvirus

HC‐Pro can act as an elicitor of R gene‐driven effector‐triggered immunity as per the gene‐for‐gene. This is the case for PVY, which induces hypersensitive responses that restrict the virus in necrotic local lesions in potato cultivars. These cultivars possess dominant resistance genes Nc_tbr_ and Ny_tbr_ (Sigvald,^1985^; Moury et al. 2011), which may recognize similar structural determinants in the central region of HC‐Pro of *PVY* ^0^ (Ny_tbr_) and *PVY* ^C^ (Nc_tbr_) strains (Tian and Valkonen 2013, 2015). Nevertheless, resistance‐breaking *PVY* isolates (*Potato virus Y* ^N^) can overcome Ny_tbr_‐mediated resistance through some residues in the C‐terminal part of the HC‐Pro (K_400_ and E_419_), causing induction of an alternative defense response of vein necrosis in tobacco infected by PVY isolates (Tribodet et al. 2005; Faurez et al. 2012). Overall, the data suggest alterations in HC‐Pro from mutations can overcome R gene‐mediated resistance, affecting functional interactions with other host factors and inducing alternative defense responses (Tian and Valkonen 2013, 2015).

In contrast to HC‐Pro, which acts as a direct elicitor of antiviral hypersensitive responses, VPg has not been reported to trigger active host immunity against viral infection. Instead, its role in defense appears specialized toward the vector; VPg has been shown to reduce aphid performance, a phenomenon likely mediated by an indirect host defense response. This mirrors the function of the 6 K1 protein, which also reduces aphid performance through the induction of phytohormone‐mediated pathways (Casteel et al. 2014; Bera et al. 2022). While VPg does not elicit active antiviral immunity, due to VPg's critical role in the viral genome translation, it has been targeted for breeding recessive resistance in plants (Moury and Verdin 2012; de Coutinho Oliveira et al. 2019). Recessive resistance is defined as a lack of susceptibility in plants. In other words, the absence of host proteins that are critical for virus infection (Fraile and García‐Arenal 2010; Mäkinen et al. 2020). A recessive resistance gene, *pvr2*, was identified in pepper plants as coding for translation initiation factor (eIF4e). VPg was found to interact directly with eIF4e, which is vital for virus translation (Kang et al. 2005; Charron et al. 2008). Mutations in the *pvr2* gene encoding for the eIF4e protein interfere with VPg binding, resulting in resistance against potyviruses (Charron et al. 2008). However, VPg mutants restore the compatible interaction with eIF4e and break the host resistance conferred by the recessive gene (Gebre‐Selassie et al. 1985). Moreover, recessive resistance was found to be highly durable for more than 50 years in pepper cultivars, suggesting most of the mutations in VPg were lethal and may have impaired their multi‐functionality (Moury and Verdin 2012).

### 4.3 Other Viruses

Several resistance genes have been identified (e.g., *Tm‐1 Tm‐2*, *Tm‐2* ^*2*^, and *Tm‐2* ^*α*^) in wild tomato species that confer resistance against tobamovirus species, including tobacco mosaic virus (TMV), ToMV, and tomato mild mottle virus (Luria et al. 2017). *Tm‐1* resistant gene from resistant tomato species encodes a protein that interacts with TMV Rep (Ishibashi et al. 2007) and prevents the formation of replication complex between Rep and membrane‐bound host proteins (TOM1, TOM2A, and ARF8) to inhibit viral replication (Ishibashi et al. 2012, 2014; Ishibashi and Ishikawa 2013). Another resistance gene, *Tm‐2*, in *S. peruvianum* confers a higher level of resistance than *Tm‐1*. The resistant gene *Tm‐2* ^*2*^ was found better in conferring resistance than *Tm‐2* (Lanfermeijer et al. 2005). The *Tm‐*2 and the *Tm‐2* ^*2*^ resistance genes are considered allelic (Pelham 1966; Young and Tanksley 1989) and encode a coiled‐coil/nucleotide binding‐ARC/LRR protein class of plant resistance (R) genes (Lanfermeijer et al. 2003). Due to coevolution, resistance‐breaking TMV incorporated two nucleotide substitutions in the Rep protein responsible for overcoming host plant resistance (Strasser and Pfitzner 2007).

Interestingly, there are a few studies that suggest the presence of an avirulent factor associated with P19 (TBSV), 2b (TAV), and NSs (TSWV) (de Ronde et al. 2013). A study on different *Nicotiana* species indicated that P19, upon agro‐infiltration, can elicit defense responses, suggesting the presence of a putative R‐protein (Angel et al. 2011). Recently, Zhu et al. (2024) have demonstrated that protein arginine methyltransferases (PRMT), PRMT6, enhance resistance to TBSV by modifying the conserved R43 and R115 residues in the viral P19 protein. This modification results in a decrease in the protein's ability to suppress silencing, which is achieved by preventing its dimerization and binding to small RNAs. Furthermore, naturally occurring PRMT6 alleles with high expression levels have been linked to increased TBSV resistance in various tomato varieties, suggesting a highly efficient avirulent factor in tomatoes.

## 5 Conclusions and Future Directions

In this review, we examine the multifunctionality of VSR proteins associated with some major viruses that affect Solanaceous hosts. The present study provides a comprehensive analysis of VSRs, emphasizing their remarkable diversity in various biological aspects. VSRs display a wide range of molecular sizes, from as small as 12 kDa to as large as 130 kDa. This size variability reflects the structural and functional diversity within the VSR family. Smaller VSRs may operate as enzymes or small protein inhibitors, while larger VSRs could serve more complex roles, possibly functioning as multi‐domain proteins capable of interacting with several host factors simultaneously. VSRs can be classified as either nonstructural or structural proteins. VSRs are found in the nucleus, others in the cytoplasm, and some can be present in both locations. VSRs interact with various cellular processes depending on their location, which could be crucial for their function in suppressing RNA silencing. For example, certain VSRs might interact with the host's silencing machinery in the cytoplasm, while others might target nuclear processes. Furthermore, VSRs can target PTGS or TGS pathways (Tables 1 and S1). In addition to their role as RNA silencing suppressors, VSRs perform a multitude of essential functions throughout the viral life cycle and in the pathogenesis of disease. Our synthesis of the existing literature on the multifunctionality of VSR proteins within an ecological context revealed several knowledge gaps, indicating potential avenues for future research. This knowledge will help us to design and improve strategies managing plant viruses and their insect vectors efficiently (Wu and Ye 2020) with the usage of fewer chemicals in agriculture, which will lead to a more sustainable and ecological plant growth (Parizad and Bera 2023).

Our review suggests a need to better understand the additional functions of VSRs and whether any are conserved across VSRs. For example, a holistic overview is missing about how VSRs modulate phytohormones such as jasmonic acid and related defense responses. Jasmonic acid is a part of the oxylipin signaling pathway and mediates volatile production and vector attraction. The downstream of the oxylipin pathway has a role in the biosynthesis of anti‐herbivore metabolites, such as green leaf volatiles, terpenoids, sesquiterpenes, and monoterpenes that function to repel herbivores or to attract natural enemies. Here, we documented how VSRs manipulate the upstream and downstream of jasmonic acid, but there are limited studies that investigated all jasmonic acid‐dependent signaling pathways simultaneously with a focus on vector attraction and repulsion during the virus life cycle. Furthermore, it would also be interesting to see if the function of VSRs changes in the presence of healthy as compared to viruliferous vectors. Exploring the possibility of the dynamic multi‐functionality of VSR to decrease virus transmission will be an important future direction.

A clear role of HC‐Pro in manipulating the ethylene hormone pathway is limited. HC‐Pro interacts with exoribonuclease, Xrn4, to neutralize host defense (Li and Wang 2018; Table S1). Xrn4 is also a component of the ethylene response pathway that is inhibited in the presence of ethylene (Olmedo et al. 2006; Potuschak et al. 2006). This suggests the ethylene hormone assists in potyvirus infection and allows HC‐Pro to be available for other purposes, as Xrn4 is suppressed by ethylene. Indeed, studies show potyvirus infection induces ethylene, which effectively neutralizes Xrn4; this may free up HC‐Pro to facilitate efficient potyvirus spread by forming the molecular bridge between the virus and the aphid stylet during transmission (Casteel et al. 2015; Bak et al. 2019). The ethylene pathway thus likely plays a central and indirect role in HC‐Pro's multifunctionality, which needs to be investigated further to allow engineering of the ethylene pathway for sustainable virus and pest management in the coming years.

The multi‐functionality of VSR proteins should also make them a good target for generating resistant plants. Moreover, the role of VSR proteins as an elicitor of defense response may lead to more durable strategies to control viruses. This is because resistance‐breaking viral strains often have fitness costs that may disrupt the multi‐functionality of a viral protein (May et al. 2020; Liu et al. 2021). Assessing potential “trade‐offs” among different virus life history traits has been conducted for various mutants in tobamoviruses (Moreno‐Pérez et al. 2016; Bera et al. 2017; Moreno‐Pérez et al. 2023; Mäkinen et al. 2020) and the same rationale can be applied for the multifunctional VSRs. VSR affects virus accumulation in systemic leaves due to its role in RNA silencing suppressor, systemic movement, and modulating phytohormones; thus, trade‐offs between the above functions should be evaluated in VSR mutants to predict the durability of resistance.

Recently, some approaches were designed to induce plant defense by using siRNA targeting VSR proteins. Begomovirus‐resistant tomato plants were developed by utilizing siRNA targeting both AC2 and AC4 ORFs. For example, Singh et al. (2015) used partial AC2 and AC4 sequences in RNAi vectors in transgenic tobacco plants to silence the AC2 and AC4 ORFs of ToLCNDV and found relatively large amounts of ToLCNDV AC2‐ and AC4‐specific transacting siRNAs. Artificial tasiRNAs also play a key role in developing resistance against AC2 and AC4 suppressors of ToLCNDV (Singh et al. 2015). All the above studies suggest VSR proteins to be a good target to develop resistant crops. These kinds of research should be encouraged for other viruses that harbor VSR proteins to produce virus‐resistant crops.

While categorizing the different functions of diverse VSR proteins from different virus genera, we found consensus functions of VSR proteins related to silencing‐suppressor activity and to modulating phytohormones and related responses that affect vector behavior. Numerous studies also showed phytohormones mediate multi‐trophic interactions consisting of herbivores, vectors, rhizobia, etc.; thus, it is tempting to speculate that VSR proteins might indirectly affect other trophic levels by modulating the phytohormone pathways (Basu et al. 2021, 2022; Lee et al. 2021). Therefore, we would like to propose focusing more on unconventional interactions that might be mediated by VSR and are missing in controlled lab environments.

## Author Contributions

**Saumik Basu** and **Sayanta Bera:** conceptualization, writing – original draft. All co‐authors (**Sourav Pal**, **Shirin Parizad**, **Pooja Malhotra**, **Trishita Ghosh**, **Clare L. Casteel**, and **David W. Crowder**): reading, review, editing, as well as approving the final manuscript.

## Funding

This research was partially supported by a US National Science Foundation award to CLC (1723926) and start‐up fund provided to SB by the University of Georgia.

## Supporting information


**Table S1:** Mode of action and multifunctionality of different viral suppressors encoded by *Solanaceae* infecting viruses.

## Data Availability

The authors have nothing to report.
