# Supplementary material for: Insights Into Virus‐Encoded RNA Silencing Suppressors Across Viral Families: A Focus on Viruses Infecting Solanaceae Crops
Source: Physiol Plant. 2026 Mar 25;178(2):e70840. doi: 10.1111/ppl.70840 (PMC13017688; doi:10.1111/ppl.70840)
Supplement: Supplementary file 1 — Table S1: Mode of action and multifunctionality of different viral suppressors encoded by Solanaceae infecting viruses. [file PPL-178-e70840-s001.docx]

**Supplementary Table 1: Mode of action and multifunctionality of different viral suppressors encoded by *Solanaceae* infecting viruses**

| Virus genus | **Susceptible crops from *Solanaceae*** | **VSR(s)** | **Molecular mechanism of silencing suppressor activity** | **Multifunctionality** | **References** |
| --- | --- | --- | --- | --- | --- |

| *Begomovirus*  satellite DNA β | Tomato, pepper, Tobacco, eggplant | V2/AV2  AC2/AL2/C2    C4/AC4  βC1 | Suppression of PTGS by inhibiting SGS3 activity,  Sequestration of ds-siRNA (21 to 24 nt) and ss-siRNAs (24 nt), suppression of TGS through interaction with AGO4 and Histone deacetylase 6. Differential interaction with RDR1.  Suppression of silencing through interaction with AGO1 and inactivation of miRNAs  Suppression of TGS mediated host defense through inactivation of SnRK1 and ADK and, interaction with host rgsCAM, H3K9me2 host methyl transferase and Su(var)3-9 homolog 4/Kryptonite (SUVH4/KYP).  Suppress silencing machinery via sequestering viral DNA through DNA binding Zn-finger motif present in them  Suppress both TGS and PTGS through sequestration of miRNAs and siRNAs and, host AGO4 protein  Suppression of both TGS and PTGS, non-specific binding with ssDNAs and dsDNAs and interference with host miRNA pathways, interaction and inactivation of SAHH (a methyl cycle maintenance enzyme), suppression of RDR6-SGS3 mediated silencing through induction of host rgsCAM. | Pre-coat protein, viral pathogenicity determinant, induce HR, interact with papain-like cysteine protease to block host defense response  Interaction with Catalase 2 enzyme for systemic spread, enhancement of SA-dependent defense signaling  Transcriptional activator, pathogenicity determinant, suppress HR induced by NSP, deregulation of host miRNAs (miR319 and miR172) responsible for various development processes, disrupts host methylation cycle, interaction between AC2 and methylation dependent host kinases inhibit transactivation of host genes through inactivating cellular transmethylation reaction.  Interaction with CNS5 and inactivation of SCF-ubiquitin related cellular defense, suppress JA mediated defense through down regulation of JA genes  Induces enhanced phloem cell division and elongation. Interact with shaggy-like kinase and interfere with brassinosteroid signaling, interact with BAM 1 and 2 and inactivation of cell to cell spread of silencing  Virus movement, pathogenicity determination, induce development abnormality in leaves through interaction with AS1, interfere with JA mediated defense response and Gibberellic acid signaling by degrading a JA-receptor (SCF^COI1^), abrogate host defense by interacting with Tm-1, interacts with WRKY20 to activate SA signaling. | Hancˇinský et al., 2020; Rojas et al., 2001; Zrachya et al., 2007; Glick et al., 2008    Sharma and Ikegami, 2009; Zhang et al., 2012; Bar-Ziv et al., 2012; Wang et al., 2014, 2018, 2019; Roshan et al., 2018, 2020; Basu et al., 2018  Karjee et al., 2008; Basu et al., 2018; Kumar and Naqvi, 2016; Hussain et al., 2007; Wang et al., 2003, 2005; Raja et al., 2008; Chung et al., 2014; Castillo-Gonzalez et al., 2015  van Wezel et al., 2002; Dong et al., 2003; Trinks et al., 2005  Lozano-Duran et al., 2011; Rosas-Diaz et al., 2016  Chellappan et al., 2005a, b; Vinutha et al., 2018; Dogra et al., 2009; Rosas-Dias et al., 2018  Cui et al., 2005; Yang et al., 2008;  Shukla et al., 2013; Li et al., 2014; Zhao et al., 2019  Kon et al., 2007; Jia et al., 2016; Voorburg et al., 2020 |
| --- | --- | --- | --- | --- | --- |
| *Tospovirus* | Eggplant, Potato, Tobacco, Pepper, Tomato, Blackberry, Tomatillo, | NSs | NSs suppress both local and non-cell autonomous systemic silencing Sequestration of siRNAs and precursor dsRNAs of miRNAs and siRNAs; local silencing through AGO1 binding via WG/GW motif. | Pathogenicity determinant Alter vector thrips performance, inactivate JA signaling through interaction with JA-regulatory components (MYC2, 3 and 4) | Takeda et al., 2002; Ocampo et al., 2018; Wu et al., 2017; Margaria et al., 2015; Schnettler et al., 2010; de Ronde et al., 2013; Hedil et al., 2015 |
| *Nepovirus* | Tomato, Potato, Blackberry, Tamarillo, | Coat protein  X4 protein | RSS activity through interaction and destabilization of AGO1 through WG/GW motif (AGO hook), contribute to temperature dependent recovery by suppressing the production of viral proteins through reduced translation by AGO1 dependent silencing (without reducing viral siRNA titer)  PTGS suppression through unknown mechanism | Encapsidation and breakdown of salicylic acid to catechol through induction of *NahG* expression | Jovel et al., 2011; Karran and Sanfacon, 2014; Ghoshal and Sanfacon, 2014  Jafarpour and Sanfacon, 2009; Jafarpour, 2010 |
| *Tombusvirus* | Eggplant, Pepper, Tomato | P19 | Very strong silencing suppressor for both local and systemic silencing.  Molecular caliper; sequester siRNA (20-22 nt) and miRNA (23 nt) duplexes with very high affinity in size specific and sequence independent manner; P19 induces expression of miR168, which intern downregulate AGO1, interfere with HEN1 mediated methylation of miRNAs to decrease endogenous miRNA stability | Interact and inactivate nucleo-cytoplasmic protein ALY; Misregulation of miR167 targeting ARF8 | Dunoyer et al., 2004; Chapman et al., 2004; Chen et al., 2008; Nasheri et al., 2011; Danielson and Pezaki, 2013; Lozsa et al., 2008; Uhrig et al., 2004; Lakatos et al., 2004 |
| *Tobamovirus* | Eggplant, Potato, Pepper, Tomato, Tamarillo | Rep | Suppressor for both local and systemic silencing; PTGS suppression by working down stream of siRNA generation  Suppress sequence specific RNA degradation by inhibiting 3’-terminal ToMV siRNA | ToMV replication, membrane binding and guanylation for 5’ capping of nascent RNA, interfere with auxin signaling | Kubota et al., 2003; Yifhar et al., 2012 |
| *Cucumovirus* | Broad host range infecting almost all plants in Solanaceae family | 2b | Sequestration of siRNA duplexes with strong affinity, suppress RDR6 mediated PTGS virus infected cells, interact with AGO1 through PAZ domain and PIWI box and with AGO4 to inhibit PTGS. | Pathogenicity determinant, induce HR, interfere with both SA, JA, ABA signaling and alter host susceptibility to herbivores | Zhang et al., 2006; Goto et al., 2007; Gonzalez et al., 2010; Hamera et al., 2012; Duan et al., 2012; Diaz-Pendon et al., 2007; Ye et al., 2009; Wang et al., 2011  Yifhar et al., 2012; Ziegler-Graff, 2020 |
| *Potyvirus* | Pepper, Potato, Tomato, Tobacco,  African eggplant,  Cape gooseberry,  Sweet pepino, tomatillo | HC-Pro  VPg | RNA silencing suppressor through different ways: Binding to vsiRNA and limiting RISC assembly by targeting multiple steps, Regulation of AGO1 function.  Mediates silencing suppressor activity through degradation of SGS3 along with RDR6 | *Autoproteolytic* activity; AGO1 recruitment and systemic infection; Inhibition of the host RNA decay mechanism through interaction with the plant exoribonuclease 4 (Xrn4).  Manifesting viral symptoms by inducing the reactive oxygen species (ROS) production; Helping viral infection by interaction with catalase 1 (CAT1) and catalase 3 (CAT3) and producing H_2_O_2_; HR induction  Suppression of SA-mediated defense responses (probable related host factors are (SA)-binding proteins (SABPs); Affecting the JA-regulated gene expression in plants.  Mediates interaction between virus and aphid resulting in efficient virus transmission, manipulation of the aphids’ biology.  Viral translation and systemic movement; Interaction with eIF4E in CAP-dependent translation of viral genome. A multiprotein complex of HC-Pro, VPg and Varicose, assist in systemic infection. | Carrington et al., 1989; Pollari et al., 2020; Li and Wang, 2018; Del Toro et al., 2017  De et al., 2018; Ivanov et al., 2016; Makinen and De, 2019; Yang et al., 2020 a, b.    Endres et al., 2010; Poque et al., 2018  Sigvald, 1985; Casteel et al., 2014  Cheng et al., 2017; Eskelin et al., 2011; Coutinho de Oliveira et al., 2019; De et al., 2020; Gebre Selassie et al., 1985; Luis-Arteaga et al., 1993; Eskelin et al., 2011 |

References

Cui, X., G. Li, D. Wang, D. Hu, and X. Zhou. 2005. “A Begomovirus DNAβ‐Encoded Protein Binds DNA, Functions as a Suppressor of RNA Silencing, and Targets the Cell Nucleus.” Journal of Virology 79, no. 17: 10764–10775.

Kon, T., P. Sharma, and M. Ikegami. 2007. “Suppressor of RNA Silencing Encoded by the Monopartite Tomato Leaf Curl Java Begomovirus.” Archives of Virology 152, no. 7: 1273–1282.

Lakatos, L., G. Szittya, D. Silhavy, and J. Burgyán. 2004. “Molecular Mechanism of RNA Silencing Suppression Mediated by p19 Protein of Tombusviruses.” EMBO Journal 23, no. 5: 876–884.

Sharma, P., and M. Ikegami. 2009. “Characterization of Signals That Dictate Nuclear/Nucleolar and Cytoplasmic Shuttling of the Capsid Protein of Tomato Leaf Curl Java Virus Associated With DNAβ Satellite.” Virus Research 144: 145–153.
